# Case Report: A Rare Syncope Case Caused by Abernethy II and a Review of the Literature

**DOI:** 10.3389/fcvm.2021.784739

**Published:** 2022-01-04

**Authors:** Xue-qin Lin, Jing-yi Rao, Yi-fei Xiang, Li-wei Zhang, Xiao-ling Cai, Yan-song Guo, Kai-yang Lin

**Affiliations:** ^1^Department of Cardiology, Shengli Clinical Medical College of Fujian Medical University, Fujian Provincial Hospital, Fuzhou, China; ^2^Fujian Provincial Key Laboratory of Cardiovascular Disease, Fujian Cardiovascular Institute, Fujian Provincial Center for Geriatrics, Fujian Clinical Medical Research Center for Cardiovascular Diseases, Fuzhou, China

**Keywords:** syncope, Abernethy malformation, congenital extrahepatic portosystemic shunt, pulmonary arterial hypertension, jaundice

## Abstract

**Background:** Abernethy malformation is an extremely rare anomaly of the splanchnic venous system, and only 2 cases that manifested as syncope had been reported previously.

**Case Presentation:** A 24-year-old male had a 15-year history of jaundice and was in long-term use of hepatoprotective drugs. He was admitted for complaint of syncope. He underwent a series of examinations and cardiac ultrasound showed that his pulmonary artery pressure was elevated. Further imaging revealed the absence of intrahepatic portal veins. His blood ammonia was significantly increased. All signs and symptoms pointed to an Abernethy diagnosis. He was finally diagnosed as having Abernethy type II. He was discharged after 17 days of in-hospital treatment with sildenafil (50 mg/day) and ornithine aspartate (20 g/day).

**Conclusion:** We now report this rare case of syncope that is caused by Abernethy malformation. As a typically pediatric disease, it was not identified in this patient until adulthood due to long-term treatment for jaundice and liver cirrhosis. Furthermore, we present a review of portosystemic shunts previously reported in the literature.

## Introduction

Abernethy malformation (AM) or congenital extrahepatic portosystemic shunt (CEPS) is a rare condition in which the porto-flow bypasses the liver and drains directly into systemic circulation. The first case of AM was described by John Abernethy in 1793 (1). AM can be classified into two types. In Type Ia, splenic and superior mesenteric veins (SMVs) drain separately into the inferior vena cava (2), and in Type Ib, draining occurs *via* a common trunk. In Type II, blood is shunted from the portal vein to inferior vena cava. Patient with this disease may develop different clinical manifestations, such as hepatic encephalopathy, pulmonary hypertension, liver tumors, or syncope (3). Syncope is very rare among these clinical manifestations, and to date, only two cases have been reported with this manifestation. We herein report a case of AM, type II, in a 24-year-old adult male presenting with syncope.

## Case Presentation

Fifteen years prior to admission, the patient presented to his primary care physician for recurrent, yellowish discoloration of the sclera and tea-colored urine. He was prescribed hepatic protectors. However, the jaundice did not resolve. Three years prior, he went through an episode of hematemesis. At that time, he was found to have liver cirrhosis with portal hypertension (PH). Consequently, he was still being treated with hepatoprotective therapy.

Three days prior to admission, he fainted suddenly as he climbed up to the third floor at his home, and he was unconscious for nearly 2 mins. After waking up, he was referred to our cardiology clinic for dizziness and headache. The physical examination showed that his sclera and skin appeared slightly “dirty-yellow” in color. On auscultation, pulmonic second heart sound (P2) was hyperactive, and there were some split sounds in the pulmonary valve area. When palpating the abdomen, the spleen could be felt at eight centimeters below the rib. He underwent a 6-min walking test, and the result was 285 meters.

Laboratory tests showed decreases in platelet counts (30 ×10^9^/L; reference range, 125–350 ×10^9^/L) and serum albumin levels (30 g/L; reference range, 40–55 g/L), and an increase in total bilirubin (82.8 μmol/L; reference range, 3.4–17.1 μmol/L). Prothrombin time (PT) was extended to 15.4 sec (reference range 9.9–12.9 sec) and the activated partial thromboplastin time (APTT) was 72.0 sec (reference range 21.5–37.5 sec). Furthermore, there was a remarkable increase in blood ammonia levels (103.9 μmol/L; reference range, 9.0–30.0 μmol/L), but no obvious signs of hepatic encephalopathy (HE). Thus, the patient's Child-Pugh score (4) was eight points (grade B), indicating a decompensated stage of liver cirrhosis. Such severe cirrhosis is not common in the young. The next step was to explore the cause of liver cirrhosis. Further laboratory tests revealed that hepatitis B surface antigen (HBsAg) was positive. The auto-immune antibodies were negative. There was a decrease in ceruloplasmin (159.0 mg/L; reference range, 200–600 mg/L) and serum copper (10.2 μmol/L; reference range, 12.7–30.3 μmol/L), while the urine copper result was negative and there was no Kayser-fleischer (KF) ring change in cornea. Since an autoimmune liver disease and congenital Wilson's disease could temporarily be ruled out, we speculated that his cirrhosis may be caused by a hepatitis B virus infection, which is one of the leading factors for liver cirrhosis (5).

**Figure 1:**
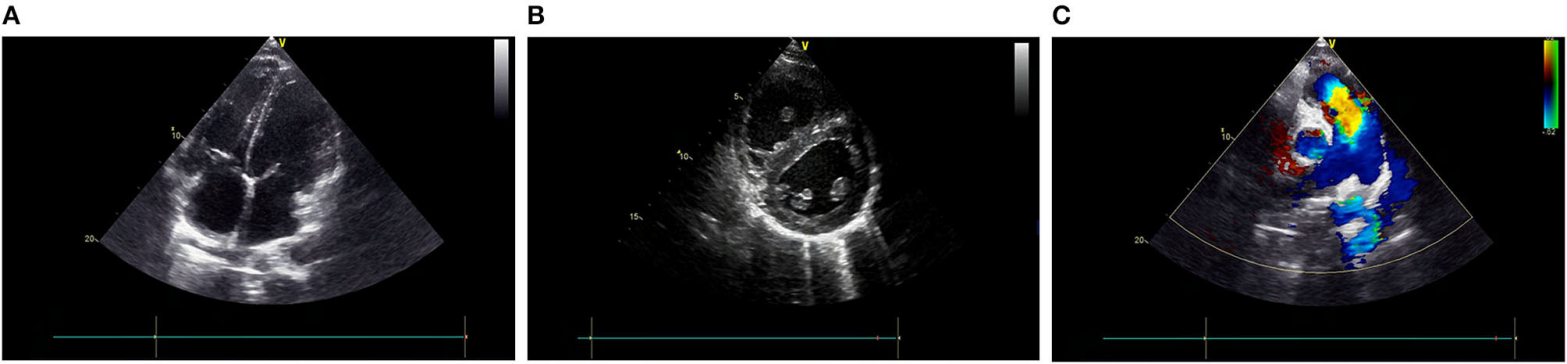
Echocardiography shows both RA and RV are significantly dilated, and the RV wall thickness is increased **(A,B)**. An enlargement of the PA and **(C)** its branches. PA, pulmonary artery; RPA, right pulmonary artery; LPA, left pulmonary artery; AO, aorta; RA, right atrium; RV, right ventricle; LA, left atrium; LV, left ventricle.

The electrocardiogram (ECG) revealed right ventricular hypertrophy. Echocardiography further confirmed that both the right atrium (RA) and right ventricle (RV) were significantly dilated (the inner diameters of the RA and RV were 4.24 and 4.31 cm, respectively, measured in the four-chamber view), and the RV wall was thickened (1.02 cm) (Figures 1A,B). Notably, there was an enlargement of the pulmonary artery and its branches (Figure 1C), and the estimated pulmonary arterial pressure (PAP) was 98 mmHg, suggesting pulmonary arterial hypertension (PAH). According to the 2018 guideline of PAH (6), we temporarily speculated that his PAH was most likely related to PH caused by cirrhosis, known as portopulmonary hypertension (POPH).

**Figure 2:**
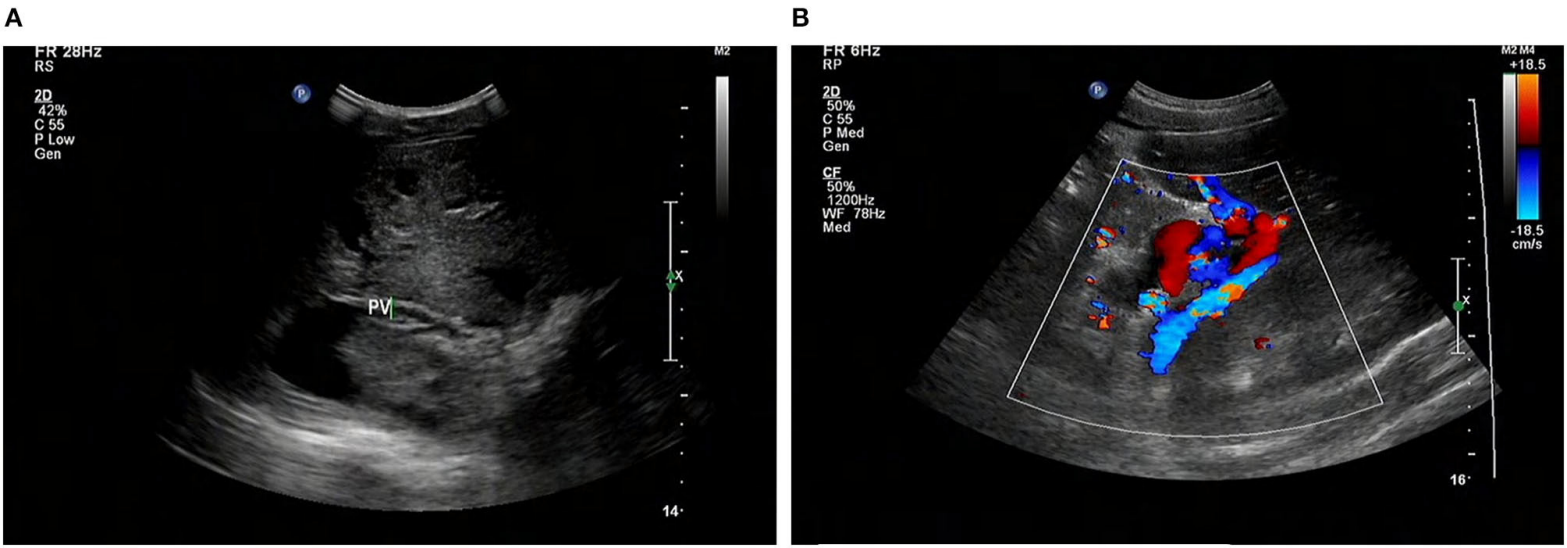
Abdominal US shows that the intrahepatic PV system is absent (arrows) **(A)**, and the GDV is tortuous and dilated **(B)**. US, ultrasonography; PV, portal vein; GDV, gastric fundus vein; STO: stomach.

**Figure 3:**
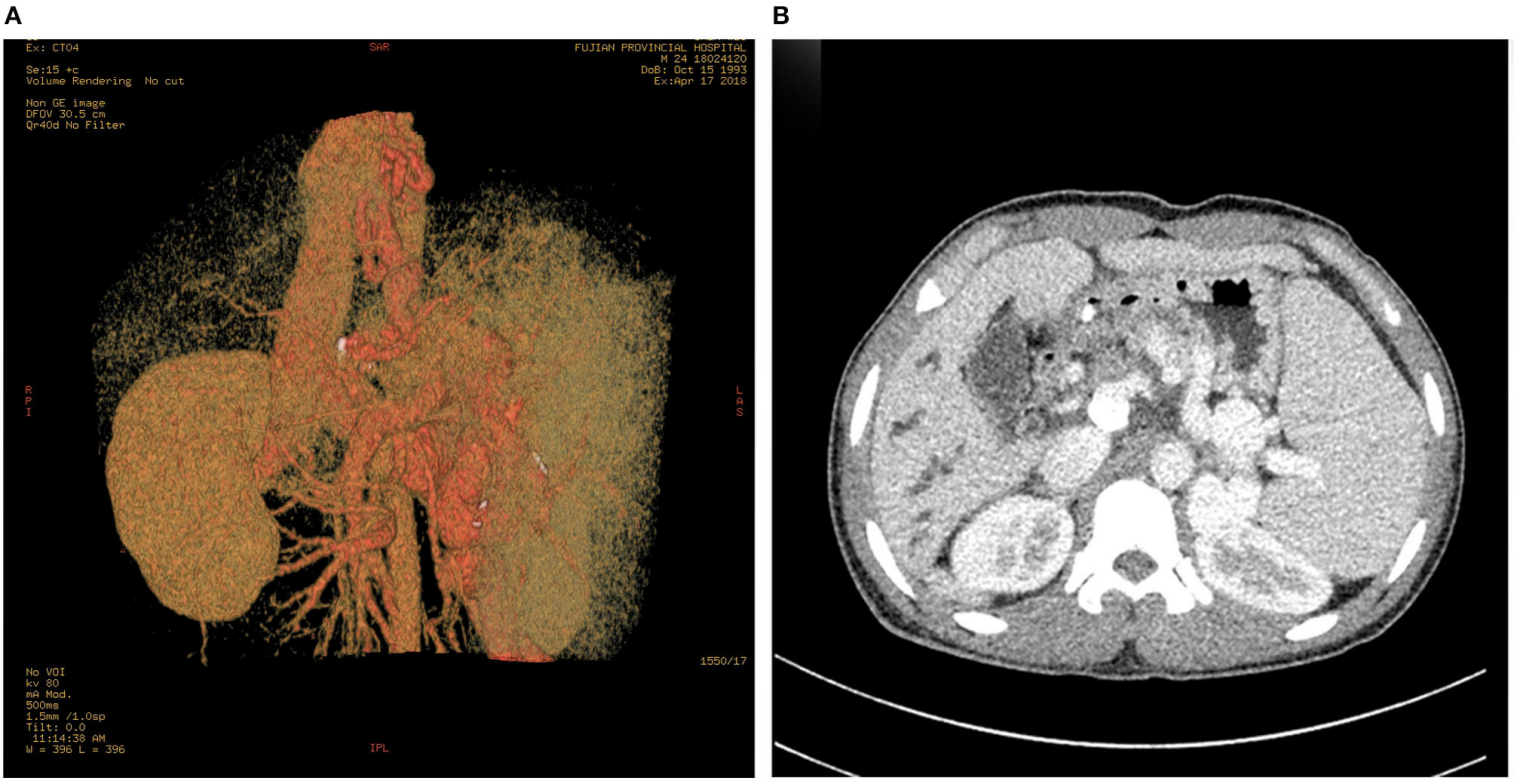
PV-CTA shows that one branch of SV flows into IVC *via* LRV **(A)**. The other branch of SV merges with SMV into PV, the intrahepatic branch of PV is slim, and the proximal trunk of PV is short and calcified **(B)**. The esophageal and gastric fundus vein **(A)**, and the SV are dilated and tortuous **(B)**. Dilatation of intrahepatic bile duct **(B)**. PV-CTA, portal vein-computed tomographic angiography; SV, splenic vein; IVC, Inferior vena cava; LRV, left renal vein; SMV, superior mesenteric vein.

**Figure 4:**
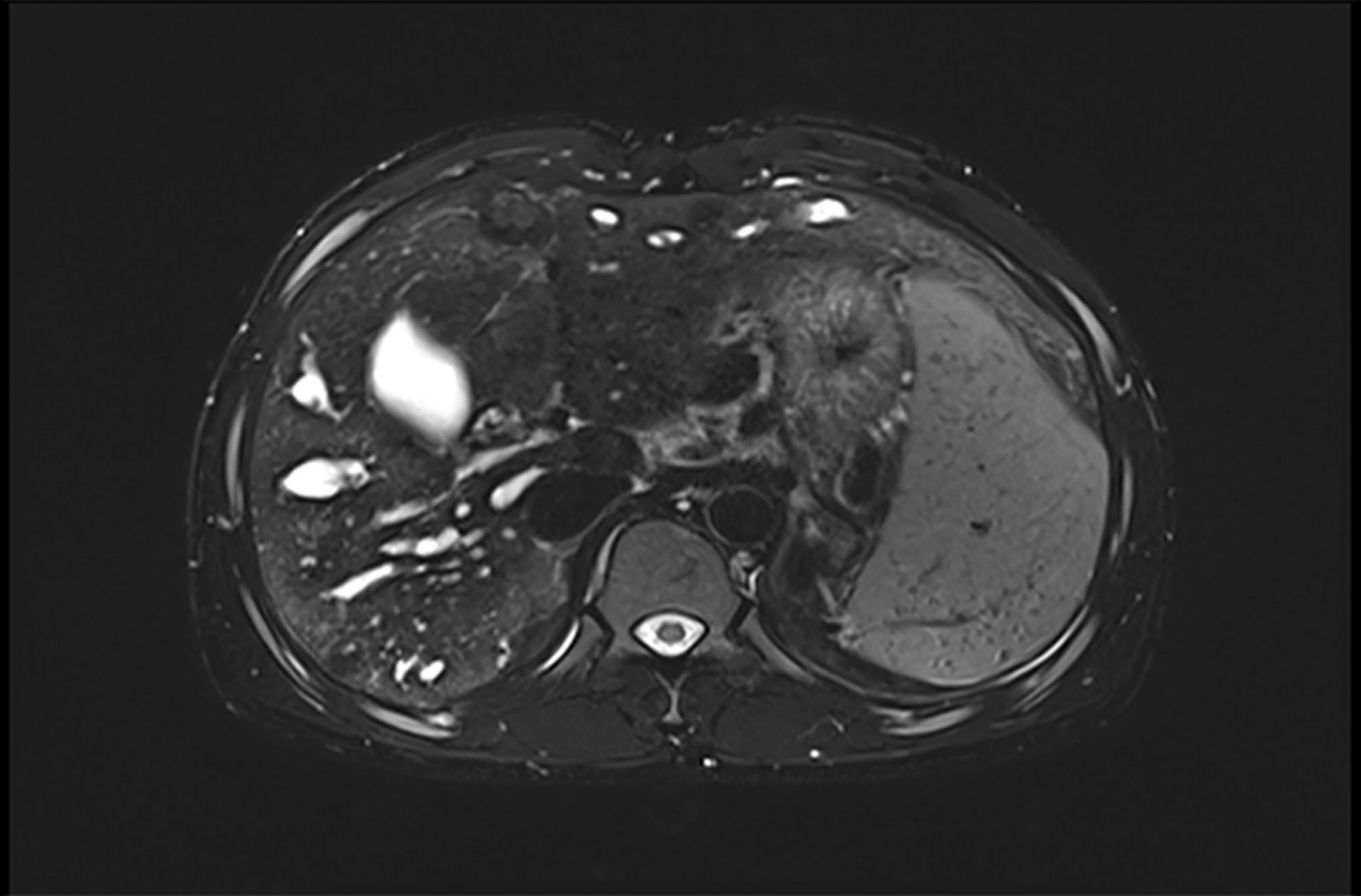
Hepatic MR shows the existence of liver nodule. MR, magnetic resonance.

At that moment, an amazing discovery was found by abdominal ultrasonography (US): his intrahepatic portal vein (PV) was absent, which seemed to be occluded (Figure 2A). To better understand this anatomic anomaly, we performed additional portal computed tomography angiography (CTA). It consistently delineated the dysplasia of the portal vein. There seemed to be two pathways for blood to flow in the main splenic vein (SV): one was in the portal vein that merged with the superior mesenteric vein and sent out a small branch into the liver (Figures 3A,B), whilst the other entered the inferior vena cava (IVC) *via* left renal vein. Also worth noting is that the hepatic artery was widened. Some regenerative nodules and abnormal hyperplasia nodules were visible. Liver magnetic resonance imaging (MRI) confirmed their existence (Figure 4).

Based on the findings described above, we established a diagnosis of CEPS (Abernethy II) combining with PAH. Due to economic limitations and personal preference, he was prescribed anti-pulmonary hypertension agents: sildenafil (50 mg/day) and ornithine aspartate (20 g/day). His condition improved, and he was discharged on day 17. More than 3 years' follow-up showed that he had no recurrence of syncope.

## Discussion

AM is a congenital vascular malformation in the liver. Imaging shows that SMV and SV were fused in this patient and partially injected into the left renal vein, and there was a small portal vein running through the liver (Figures 2, 3). According to the classification proposed by Morgan and Superina (2), these features are classified as AM type II. To the best of our knowledge, AM rarely presents with syncope, and our patient is the first case of a male with CEPS (Abernethy II) showing syncope.

Although AM is less common, it has been gradually acknowledged by clinicians. Its prognosis is poor (7). We did a systemic review of AM. The literature research process is shown in Supplementary Figure 1. We searched PubMed, Web of Science, and Embase and Cochrane databases using the search terms “Patent ductus venosus,” “Abernethy malformation,” “congenital portosystemic shunt,” “extrahepatic portosystemic shunt,” “congenital portosystemic venous shunt,” “congenital porto-caval shunt,” “congenital porto-caval shunts,” “congenital porto-systemic shunt,” “congenital porto-systemic shunts,” “congenital portocaval shunt,” “congenital portocaval shunts,” “congenital portosystemic shunts,” “portocaval shunt, congenital,” and “portosystemic shunt, congenital,” limited to the English language. From the resulting 451 articles, the title, abstract, and the article text were reviewed. Articles were excluded if they provided insufficient data or discussed patients with clinical problems outside of our target group (e.g., arteriovenous, or surgical shunts, or shunts caused by cirrhosis or portal vein thrombosis). In total, 703 patients' data from 451 articles were extracted and analyzed (see Table 1). The minimal information required to include a patient in the analysis was full-text version of an article and congenital extrahepatic shunts.

**Table 1:** Literature review.

**Classification**		**Number**	**Gender**			**Age (years)**			**Hepatic nodules**					**PAH**	**PH**	**HE**	**HPS**	**Syncope**
			**Male**	**Female**	**Unknown**	** <18**	**≥18**	**Unkown**	**Adenoma**	**HCC**	**FNH**	**Hemangioma (8)**	**Unclassified**					
I	Ia	8	4	4	0	7	1	0	0	1	1	0	1	1	0	0	0	0
	Ib	109	50	51	8	73	35	1	10	12	20	1	17	17	2	5	9	1
	Unknown	53	26	19	8	27	8	18	1	4	4	0	14	3	0	3	5	0
II		243	131	81	31	152	46	45	7	4	12	0	23	40	4	12	23	1
Unknown		290	67	69	154	106	26	158	2	4	3	0	17	43	18	14	45	0
Total amount		703	278	224	201	365	116	222	20	25	40	1	72	104	24	34	82	2

*HCC, hepatic cellular carcinoma; FNH, focal nodular hyperplaisa; PAH, pulmonary arterial hypertension; PH, portal hypertension; HE, hepatic encephalopathy; HPS, hepatic pulmonary syndrome*.

After analyzing all the reported cases (Table 1), excluding the population that lack the information about gender, it seems to be more common in men (1.2:1), especially type II (1.6:1). More than half of all published cases were diagnosed under the age of 18. Similarly, a report was found of a 15-year-old female patient with AM type Ib published 2 years prior (9). However, different from the demographic data collected in our review of the literature, the patient in this report was the first to be diagnosed with CEPS at 24 years old. Complications occurred in some patients (Table 1). In the reported cases, more than 20% of the patients had liver nodules, such as liver adenoma, hepatocellular carcinoma, focal nodular hyperplasia (FNH), and liver hemangioma. In the current case, abdominal CTA and liver MR also confirmed the presence of liver nodules. The proportions of cases with PAH and PH are 14.79% and 3.41%, respectively. In our case, the pulmonary hypertension may be related to the shunting of vasoactive substances in the portal vein. In recent years there were some incidental cases diagnosed with atypical clinical manifestations. The clinical manifestations of AM are diverse, and there are also some rare clinical manifestations, such as those with nephrotic syndrome (10) as the primary manifestation, or endocrine diseases such as hyperinsulinemia (11–13). In addition to the atypical manifestations above, there were two patients previously reported to experience syncope (11, 14).

Of the two syncope cases, the first was a 42-year-old female patient, diagnosed with AM type II (14). The other was a 17-year-old female with AM Ib, manifesting as repeated syncope (11). Due to abnormal liver blood perfusion (15), liver nodules were reported in both patients (11, 14). Our patient also had this abnormality. All three syncope patients had some of the same characteristics including high blood ammonia, PAH and PH. Our patient is also quite different from the other syncope cases (11, 14). Both of the other two reported cases were women. Moreover, the one with the 42-year-old female did not suffer from liver cirrhosis. The other case is AM type Ib, combined with endocrine complications (pigmentation-acanthus nigra and irregular menstruation). Meanwhile, the 17-year-old female had evidence of HE (11), which is probably related to syncope. Our patient had not met the criteria for an encephalopathy diagnosis (16). The author of the 17-year-old female's case report believes that the patient's disturbance of consciousness may have been caused by hyperammonemia or hypoglycemia. In our case, his blood ammonia was elevated, possibly caused by liver cirrhosis and a portosystemic shunt. According to the ammonia-poisoning theory (17), we speculated that there might be a disturbance of consciousness caused by elevated blood ammonia in our case. As far as we know, syncope is not uncommon in patients with liver cirrhosis (11, 18), even in the absence of HE. Blood ammonia can be checked to assist in diagnosis. At the same time, our patient had severe pulmonary hypertension, which may have led to cardiogenic syncope. PAH could induce decreased preload of the heart and low encephalic perfusion, promoting the onset of syncope.

In terms of treatment, AM type I patients can only be cured by liver transplantation (19). Type II patients can obtain a very good curative effect by surgically clamping the drainage tube (20). Our patient declined shunt-closure therapy for economical reasons and personal preference. Instead, he only took drugs that were hepatoprotective, and reduced pulmonary hypertension and blood ammonia. After more than 3 years' follow-up, our patient is still alive and has not fainted. This is an extremely rare case of an atypical, male-AM type II with syncope.

## Conclusion

In summary, we reported a case of AM type II, manifesting as syncope (see Supplementary Figure 2. Medical history). We further reviewed 703 cases of CEPS. Clinicians should note that syncope may develop in these patients, possibly due to high blood ammonia or PAH caused by CEPS.

## Data Availability Statement

The raw data supporting the conclusions of this article will be made available by the authors, without undue reservation.

## Ethics Statement

Written informed consent was obtained from the individual (s) for the publication of any potentially identifiable images or data included in this article. Patient consent form was read and signed by the patient.

## Author Contributions

X-qL was responsible for the clinical design and conceptualization. J-yR, Y-fX, and X-lC were involved in the acquisition of the clinical data. Y-sG and K-yL conducted the clinical diagnosis. X-qL, Y-fX, and L-wZ analyzed and interpreted the data. X-qL and J-yR wrote the manuscript. All authors discussed, read, and approved the submission of this manuscript to the journal.

## Funding

This study was funded by a grant from the Joint Funds for the Innovation of Science and Technology, Fujian Province (Grant Number: 2018Y9097), high-level hospital foster grants from Fujian Provincial Hospital, Fujian Province, China (Grant Number: 2020HSJJ05).

## Conflict of Interest

The authors declare that the research was conducted in the absence of any commercial or financial relationships that could be construed as a potential conflict of interest.

## Publisher's Note

All claims expressed in this article are solely those of the authors and do not necessarily represent those of their affiliated organizations, or those of the publisher, the editors and the reviewers. Any product that may be evaluated in this article, or claim that may be made by its manufacturer, is not guaranteed or endorsed by the publisher.

## Abbreviations

AM: Abernethy malformation
CEPS: Congenital extrahepatic portosystemic shunt
SMV: superior mesenteric vein
PH: portal hypertension
PT: Prothrombin time
APTT: activated partial thromboplastin time
HE: hepatic encephalopathy
P2: pulmonic second heart sound
HBsAg: hepatitis B surface antigen
KF: Kayser-fleischer
ECG: electrocardiogram
RA: right atrium
RV: right ventricle
PAP: pulmonary arterial pressure
POPH: portopulmonary hypertension
US: abdominal ultrasonography
PV: portal vein
CTA: computed tomography angiography
SV: splenic vein
IVC: inferior vena cava
MRI: magnetic resonance imaging
FNH: focal nodular hyperplasia.
