# Supplementary material for: Case Report: A Rare Syncope Case Caused by Abernethy II and a Review of the Literature
Source: Front Cardiovasc Med. 2022 Jan 4;8:784739. doi: 10.3389/fcvm.2021.784739 (PMC8764447; doi:10.3389/fcvm.2021.784739)
Supplement: Supplementary file 1 [file Data_Sheet_1.PDF]

Supplementary Material

1.1 Supplementary Figures 1.

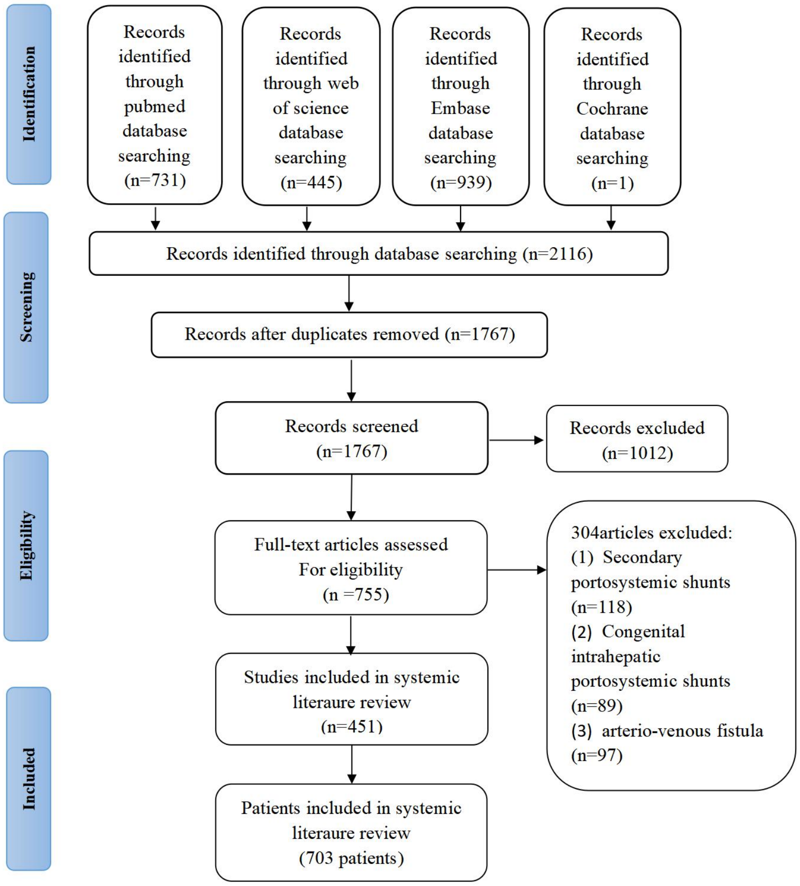

Supplementary Figure 1. literature research process. We searched PubMed, Web of Science, Embase and Cochrane databases. After passing the inclusion and exclusion criteria, 451 articles were finally included. The inclusion criteria are full text and congenital extrahepatic portosystemic shunt. The exclusion criteria are arteriovenous, or surgical shunts, or shunts caused by cirrhosis or portal vein thrombosis. In these selected articles, the title, abstract, and the article text were reviewed. In total, 703 patients’ data from 451 articles were extracted and analyzed.

1.2 Supplementary Figures 2.

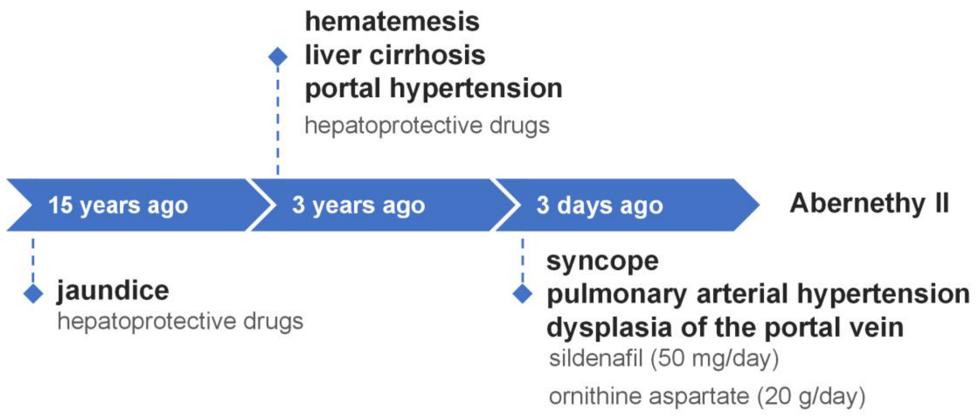

Supplementary Figure 2. Medical history. A 24-year-old male had a 15-year history of jaundice and was in long-term use of hepatoprotective drugs. Three years prior, he had an episode of hematemesis. At that time, he was found to suffer liver cirrhosis with portal hypertension. However, he was still being treated with hepatoprotective therapy. Three days prior, he fainted suddenly and lost consciousness for two minutes. Then he was referred to our cardiology clinic for dizziness and headache after recovery from syncope. The cardiac ultrasound showed that his pulmonary artery pressure was elevated. Further imaging revealed the absence of intrahepatic portal veins. All signs and symptoms were pointed to an Abernethy diagnosis. He was finally diagnosed as Abernethy type II and was discharged after treatment with sildenafil (50 mg/day) and ornithine aspartate (20 g/day).
